# Mathematical learning deficits originate in early childhood from atypical development of a frontoparietal brain network

**DOI:** 10.1371/journal.pbio.3001407

**Published:** 2021-09-30

**Authors:** Ulrike Kuhl, Sarah Sobotta, Legascreen Consortium, Michael A. Skeide

**Affiliations:** 1 Research Group Learning in Early Childhood, Max Planck Institute for Human Cognitive and Brain Sciences, Leipzig, Germany; 2 Machine Learning Group, Faculty of Technology, Bielefeld University, Bielefeld, Germany; Universität Tübingen, GERMANY

## Abstract

Mathematical learning deficits are defined as a neurodevelopmental disorder (dyscalculia) in the International Classification of Diseases. It is not known, however, how such deficits emerge in the course of early brain development. Here, we conducted functional and structural magnetic resonance imaging (MRI) experiments in 3- to 6-year-old children without formal mathematical learning experience. We followed this sample until the age of 7 to 9 years, identified individuals who developed deficits, and matched them to a typically developing control group using comprehensive behavioral assessments. Multivariate pattern classification distinguished future cases from controls with up to 87% accuracy based on the regional functional activity of the right posterior parietal cortex (PPC), the network-level functional activity of the right dorsolateral prefrontal cortex (DLPFC), and the effective functional and structural connectivity of these regions. Our results indicate that mathematical learning deficits originate from atypical development of a frontoparietal network that is already detectable in early childhood.

## Abstract 2

Longitudinal neuroimaging of 3-6-year-old children reveals a predisposition for dyscalculia in early childhood originating from altered spontaneous activity, functional interaction and structural connectivity of a frontoparietal brain network.

## Introduction

Developmental learning disorder with impairment in mathematics (ICD-11 6A03.2 https://icd.who.int, hereafter: dyscalculia) occurs in as much as 3% to 7% of the population [1]. Affected individuals suffer from devastating consequences for educational opportunity, psychosocial well-being, mental health, and professional achievement [2,3].

Like other developmental disorders such as attention-deficit/hyperactivity disorder and autism spectrum disorder, dyscalculia is classified as a neurodevelopmental disorder. Previous work in 11- to 12-year-old children identified hemodynamic underactivations of parietal and prefrontal cortices during numerosity comparison as the functional neural correlates of dyscalculia [4]. Additional observation and intervention studies and meta-analyses in children consistently identified these regions as a canonical mathematical information processing network connected by the superior longitudinal fasciculus (SLF) [5–8].

Compared to other neurodevelopmental learning disorders, like dyslexia, the brain basis of dyscalculia is still strongly understudied [9]. In particular, the current lack of longitudinal studies following children before they undergo mathematical instruction limits our understanding of the actual developmental origins of dyscalculia. Such preinstruction studies are considered as the gold standard approach to disentangle potential predispositions for developing a learning disorder from the qualitatively and quantitatively different learning experience that affected individuals have [10].

In the present study, a sample of 3- to 6-year-old children without formal mathematical learning experience underwent functional and structural magnetic resonance imaging (MRI) and was then followed until the age of 7 to 9 years (second grade in school) when they were comprehensively assessed for mathematical and other cognitive abilities. We identified 15 children who developed dyscalculia according to an operational definition (1 standard deviation [SD] below the math performance of a reference population). These children were matched to 15 typically developing children to minimize differences in age, sex, handedness, maternal education, language ability, nonverbal intelligence quotient (IQ), and verbal short-term memory. A priori power analyses based on effect sizes of previously published studies revealed power levels of 0.96 to 0.99 for our sample.

Our starting point was to explore group differences in spontaneous resting-state hemodynamic activity at the regional level (amplitude of low-frequency fluctuations and regional homogeneity) and at the network level (degree centrality). Although the consistent results reported in the literature allowed us to generate anatomically specific hypotheses, we did not initially constrain our analysis to predefined regions of interest. Instead, to account for the lack of groundwork on the specific population of young unschooled children, we conducted anatomically unbiased whole-brain analyses. To maximize the sensitivity and specificity of the group classification, we ran multivariate pattern analyses following a searchlight-based approach. Post hoc, we also examined whether the classification results were driven by a particular component of mathematical cognition (visuospatial numerosity detection and calculation problem solving). The next step was an effective functional connectivity (Granger causality) analysis designed to find out whether causal interactions between nodes of the mathematical processing network differed between children who developed dyscalculia and typical controls. Finally, based on diffusion-weighted MRI data, we also reconstructed white matter (WM) fiber pathways connecting these nodes and tested for group differences in streamline density.

Following the available results for older children already suffering from dyscalculia, we expected significant functional and structural differences compared to controls in the canonical mathematical processing network comprising parietal and prefrontal cortices and their structural connection via the SLF.

## Results

### A priori power analyses

The statistical power of our resting-state fMRI analysis was estimated based on a previous study comparing 7- to 11-year-old schoolchildren with dyscalculia (*n* = 15) with typical controls (*n* = 16) [11]. In this study, differences with respect to the coupling of hemodynamic activation time courses revealed a large effect size (Cohen’s *d* = 1.44) in the right posterior parietal cortex (PPC). According to an a priori power analysis based on the difference between 2 independent means, these effects can be replicated at a power level of 0.96 assuming a corrected threshold of *P* < 0.05 in the present sample of children with dyscalculia (*n* = 15) and typical controls (*n* = 15).

We conducted a similar a priori power analysis for our diffusion-weighted MRI analysis based on a previous study comparing 8- to 11-year-old schoolchildren with dyscalculia (*n* = 15) with typical controls (*n* = 15) [12]. The effect size of the reported differences in WM connectivity of the right SLF was large (Cohen’s *d* = 1.50). In the current sample, this effect can be replicated at a power level of 0.99 given a corrected threshold of *P* < 0.05.

### Sample characteristics

Children who developed dyscalculia did not differ from typically developing matched controls in terms of age, sex, handedness, maternal education, language ability, nonverbal IQ, and verbal short-term memory (all *d* < 0.69, all *P* > 0.517, assessed at baseline in kindergarten). However, the math ability of children who developed dyscalculia was significantly lower compared to typical controls (visuospatial numerosity detection: *d* = 2.85; calculation problem solving: *d* = 2.29; all *P* < 0.001, assessed at follow-up in school). All sample characteristics are specified in Table 1.

**Table 1:** Sample characteristics.

	Dyscalculia group	Control group	Comparison^9^
Age^1^ (mean ± SD^2^ | min–max)	4 y 11 m ± 8 m 3 y 11 m–6 y 1 m	5 y 0 m ± 9 m 3 y 11 m–6 y 0 m	*d* = 0.24 | *P* = 0.699
Sex (male | female)	4 | 11	6 | 9	*d* = 0.69 | *P* = 0.699
Handedness (LQ^3^ mean ± SD | right | ambidextrous | left)	71 ± 35 14 | 0 | 1	65 ± 40 13 | 1 | 1	*d* = 0.04 | *P* = 0.899
Maternal education^4^ (mean ± SD | min–max)	4.47 ± 1.19 3–6	4.27 ± 1.39 3–7	*d* = 0.20 | *P* = 0.591
Language ability^5^ (mean ± SD | min–max)	56.13 ± 24.17 0–100	57.07 ± 5.74 49–68	*d* = 0.24 | *P* = 0.517
Nonverbal IQ^6^ (mean ± SD | min–max)	96.73 ± 10.44 76–111	98.87 ± 11.91 81–125	*d* = 0.19 | *P* = 0.606
Verbal short-term memory^7^ (mean ± SD | min–max)	9.13 ± 2.30 6–15	8.80 ± 2.24 6–13	*d* = 0.24 | *P* = 0.525
Math ability^8^ (mean ± SD | min–max)	9.40 ± 4.78 1–16	67.93 ± 26.45 31–99	

^1^ Age in years (y) and months (m) at which children underwent MRI.

^2^ Standard deviation.

^3^ LQ (custom version of the Edinburgh Handedness Inventory adapted for children).

^4^ Combined score of mother’s school education (4-point scale: no degree: 0 points; German “Abitur” (high school diploma/A level): 3 points) and vocational qualification (5-point scale: no qualification: 0 points; German “Habilitation” (postdoctoral academic qualification): 4 points) (self-designed custom questionnaire).

^5^ Standard scores (T) with mean and SD = 50 ± 10 (German sentence comprehension test for children (TSVK)).

^6^Standard scores with mean and SD = 100 ± 15 (WPPSI-III).

^7^ Raw scores of a number sequence recall task, sequence length increases every 3 items from 2 to maximally 9 until all 3 items of a length are recalled incorrectly, children receive a point for each correctly recalled number sequence (K-ABC).

^8^ Percentile ranks (HRT); group comparison statistic not reported to avoid circularity.

^9^Effect size (Cohen’s *d)* | *P* value (categorical data: Fisher exact test, normally distributed continuous data: Student *t* test, not normally distributed continuous data: Wilcoxon signed-rank test).

HRT, Heidelberg calculation test; IQ, intelligence quotient; K-ABC, Kaufman Assessment Battery for Children; LQ, laterality quotient; MRI, magnetic resonance imaging; WPPSI-III, Wechsler Preschool and Primary Scale of Intelligence.

### Regional functional activity

**Fig 1:**
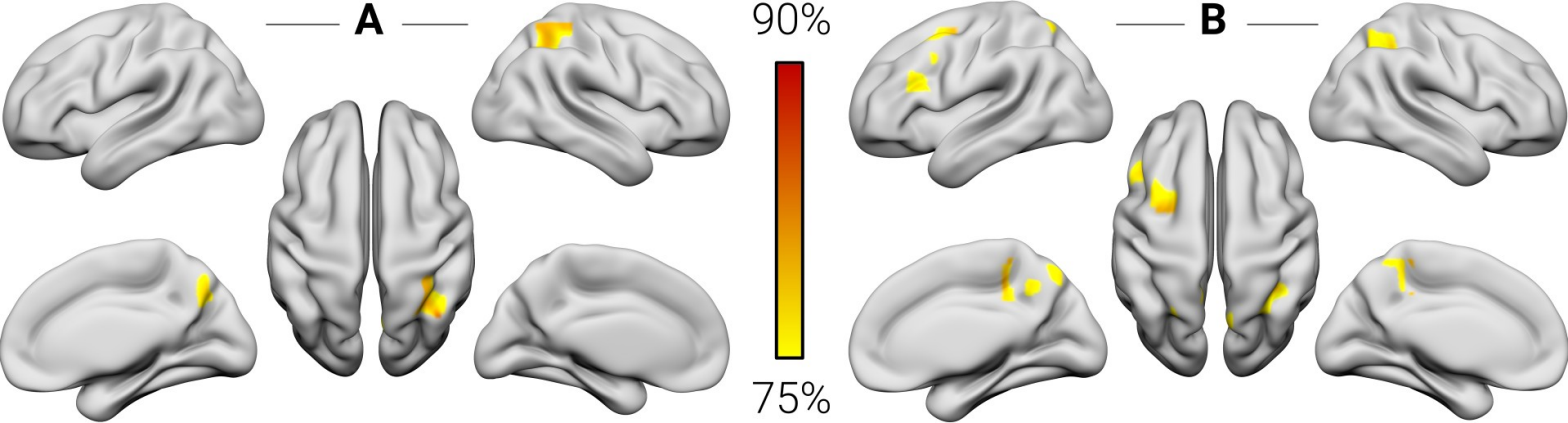
Regional functional classification results. The color bar indicates the peak accuracy for the classification of children with dyscalculia versus typical controls (*P* < 0.001, corrected by permutation testing) based on the amplitude of low-frequency fluctuations and the regional homogeneity of the signals. The numerical data used in this figure are included in S1 Data.

As a first step, we explored at the whole-brain level whether regional hemodynamic activity in children who went on to develop dyscalculia differed from typically developing children. Our classifier was able to significantly distinguish between the groups based on the amplitude of low-frequency fluctuations and the regional homogeneity of the signals. Peak classification accuracy for the amplitude of low-frequency fluctuations was 86.67% in the right PPC and 80% in the right precuneus (PC) (*P* < 0.001, corrected by permutation testing) (Fig 1A, Table 2). Peak classification accuracy for regional homogeneity was 86.67% in the right PC and the left dorsolateral prefrontal cortex (DLPFC), 83.34% in the right and left PPC and the left DLPFC, and 76.67% in the left frontal pole (*P* < 0.001, corrected by permutation testing) (Fig 1B, Table 2).

**Table 2:** Regional functional classification results.

**Amplitude of low-frequency fluctuations**								
**Size (voxels)**	**Size (mm** ^**3**^ **)**	**Peak accuracy (%)**	**Sensitivity at peak**	**Specificity at peak**	**MNI coordinates xyz**			**Anatomical location**
25	900	86.67	86.67	86.67	+30	−54	+44	PPC
27	972	80.00	73.34	86.67	+12	−63	+46	PC
**Regional homogeneity**								
**Size (voxels)**	**Size (mm** ^**3**^ **)**	**Peak accuracy (%)**	**Sensitivity at peak**	**Specificity at peak**	**MNI coordinates xyz**			**Anatomical location**
40	1,440	86.67	86.67	86.67	+12	−51	+45	PC
32	1,152	86.67	86.67	86.67	−24	+12	+41	DLPFC
29	1,044	83.34	80.00	86.67	+30	−45	+46	PPC
29	1,044	83.34	80.00	86.67	−17	−62	+42	PPC
27	972	83.34	80.00	86.67	−45	+24	+25	DLPFC
22	792	76.67	66.67	86.67	−27	+54	−02	Frontal pole

DLPFC, dorsolateral prefrontal cortex; MNI, Montreal Neurological Institute; PC, precuneus; PPC, posterior parietal cortex.

### Network-level functional connectivity

**Fig 2:**
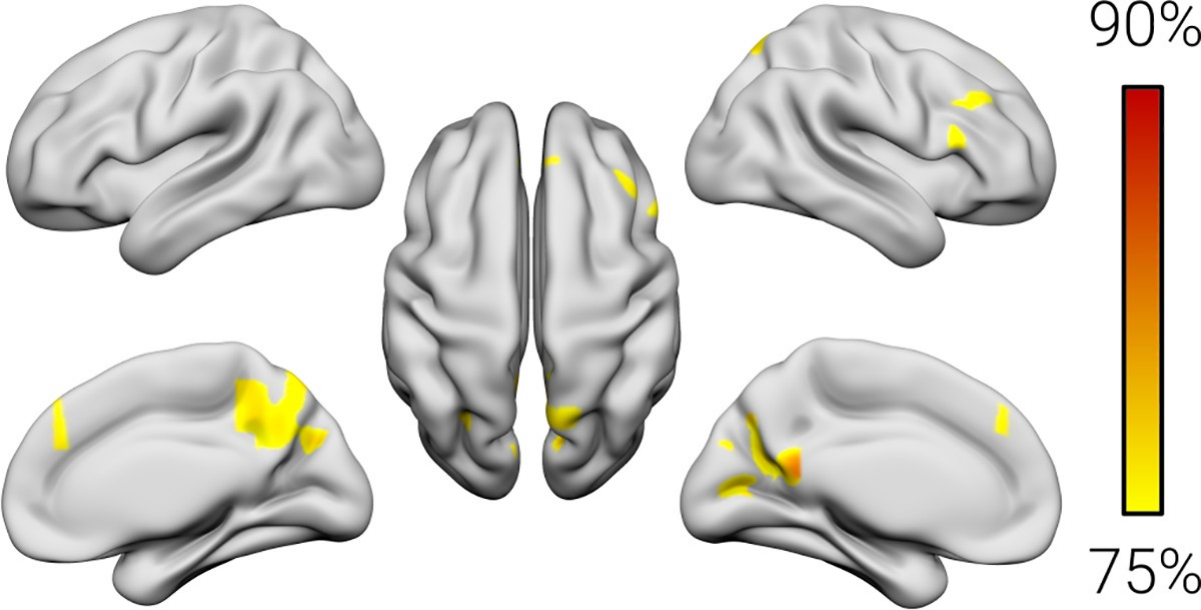
Network-level functional classification results. The color bar indicates the peak accuracy for the classification of children with dyscalculia versus typical controls (*P* < 0.001, corrected by permutation testing) based on whole-brain functional connectivity (degree centrality). The numerical data used in this figure are included in S2 Data.

Our next step was to explore whether also the network-level hemodynamic activity of the future dyscalculia group differed from controls. To this end, we examined the functional connectivity between each voxel and all other voxels of the brain without predefining seed regions by computing whole-brain degree centrality maps. These maps revealed a peak classification accuracy of 86.67% in the right orbitofrontal cortex and 83.34% in the right DLPFC, the left PC and the right cuneus (*P* < 0.001, corrected by permutation testing) (Fig 2, Table 3).

**Table 3:** Network-level functional classification results.

Size (voxels)	Size (mm^3^)	Peak accuracy (%)	Sensitivity at peak	Specificity at peak	MNI coordinates xyz			Anatomical location
31	1,116	86.67	93.34	80.00	+03	+24	−18	Orbitofrontal cortex
68	2,448	83.34	80.00	86.67	+45	+27	+30	DLPFC
46	1,656	83.34	93.34	73.34	−15	−57	+25	PC
44	1,586	83.34	86.67	80.00	+18	−72	+30	Cuneus

DLPFC, dorsolateral prefrontal cortex; MNI, Montreal Neurological Institute; PC, precuneus.

### Functional dissociation of cognitive components

The test used to determine dyscalculia combines 2 fundamental components of mathematical cognition, namely, visuospatial numerosity detection and calculation problem solving. Accordingly, we wanted to find out whether the reported classification results were driven by region-specific associations with a particular component. Following a recent meta-analysis of mathematical processing in children, we focused on the cluster in the right PPC, a region that was most strongly associated with numerosity, and on the clusters in the left and right DLPFC, a region that was most strongly associated with calculation [5]. We found that a significantly higher proportion of variance in visuospatial numerosity detection (*R*^*2*^ = 0.20) compared to calculation problem solving (*R*^*2*^ = 0.05) was explained by the amplitude of low-frequency fluctuations of the right PPC (Cohen’s *d* = 0.90, *P* = 0.015, family-wise error corrected), but not by the regional homogeneity of the right PPC (Cohen’s *d* = 0.67, *P* = 0.061, family-wise error corrected). Additionally, a significantly higher proportion of variance in calculation problem solving (*R*^*2*^ = 0.64) compared to visuospatial numerosity detection (*R*^*2*^ = 0.57) was explained by the degree centrality of the right DLPFC (Cohen’s *d* = 0.49, *P* = 0.017, family-wise error corrected), but not by the regional homogeneity of the left DLPFC (Cohen’s *d* = 0.26, *P* = 0.306).

### Effective functional connectivity

**Fig 3:**
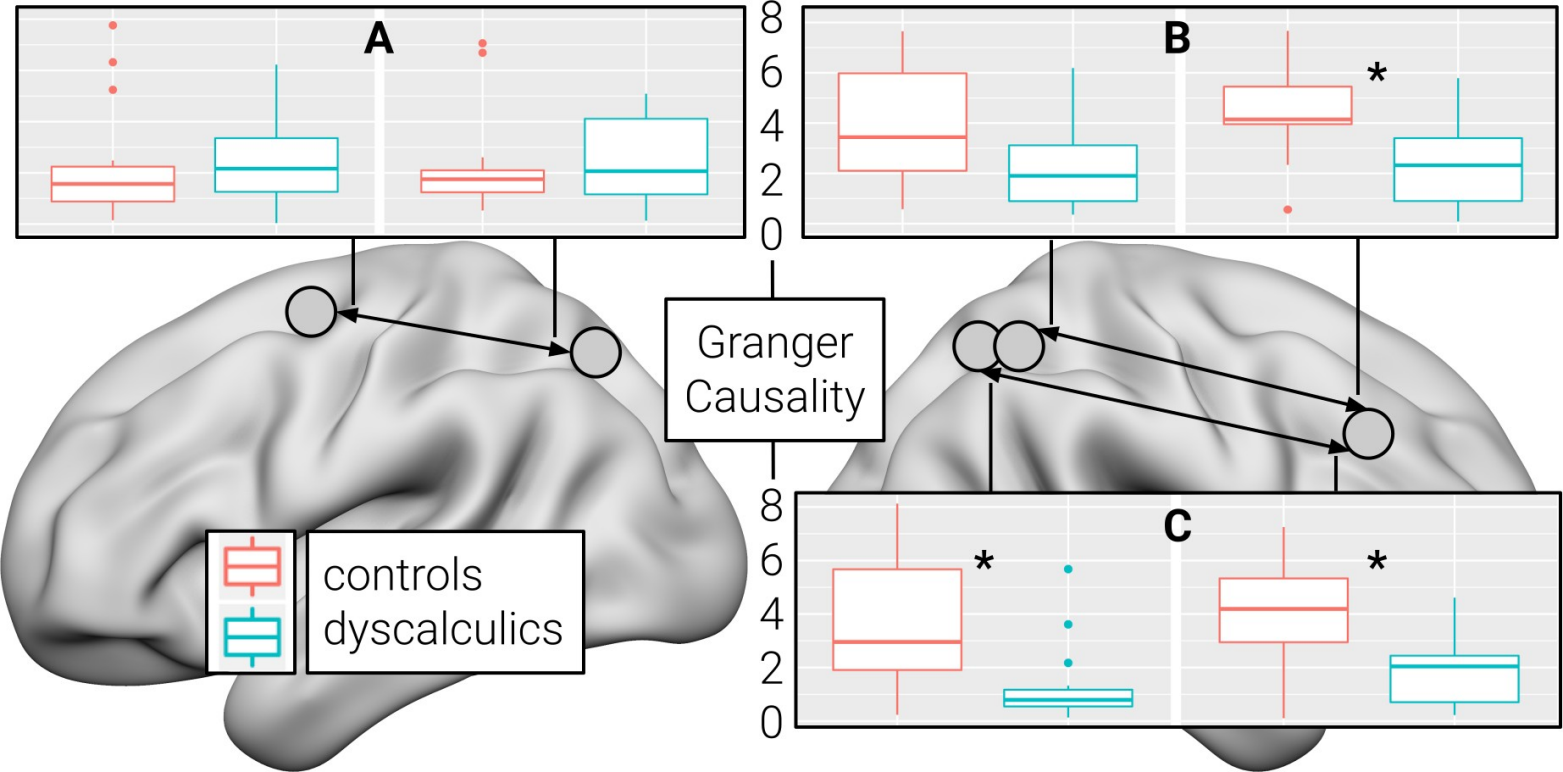
Effective connectivity differences. Granger causality indices for pairs of regions of interest derived from (**A**) the regional homogeneity of the left PPC and the left DLPFC, (**B**) the amplitude of low-frequency fluctuations of the right PPC and the degree centrality of the right DLPFC, and (**C**) the regional homogeneity of the right PPC and the degree centrality of the right DLPFC. Arrows indicate the direction of the influence of one hemodynamic time series onto the other. Vertical lines at the top and the bottom of the bars depict the SD. Horizontal lines within the bars represent the group median. Dots denote single cases that are more than 1.5 SDs away from the group mean. Asterisks indicate significant differences between children with dyscalculia and controls at a threshold of *P* < 0.05 (family-wise error corrected). The numerical data used in this figure are included in S3 Data. DLPFC, dorsolateral prefrontal cortex; PPC, posterior parietal cortex.

Building on these results, we also investigated whether the causal interaction between the PPC and the DLPFC differed between future dyscalculics and typical controls. To this end, we compared Granger causality indices quantifying the linear directional influence of one hemodynamic time series onto the other. Compared to children who developed dyscalculia, control children showed a significantly stronger influence of the right PPC on the right DLPFC (pair of clusters derived from the regional homogeneity of the right PPC and the degree centrality of the right DLPFC: Cohen’s *d* = 1.41, *P* = 0.004, family-wise error corrected; pair of clusters derived from the amplitude of low-frequency fluctuations of the right PPC and the degree centrality of the right DLPFC: Cohen’s *d* = 1.11, *P* = 0.030, family-wise error corrected) and partly also in the opposite direction (PPC cluster derived from regional homogeneity: Cohen’s *d* = 1.35, *P* = 0.013, family-wise error corrected; PPC cluster derived from the amplitude of low-frequency fluctuations: Cohen’s *d* = 0.85, P = 0.186, family-wise error corrected). No significant differences in both directions were observed for the clusters derived from the regional homogeneity of the left PPC and the left DLPFC (all Cohen’s *d* < 0.26) (Fig 3).

### Structural connectivity

**Fig 4:**
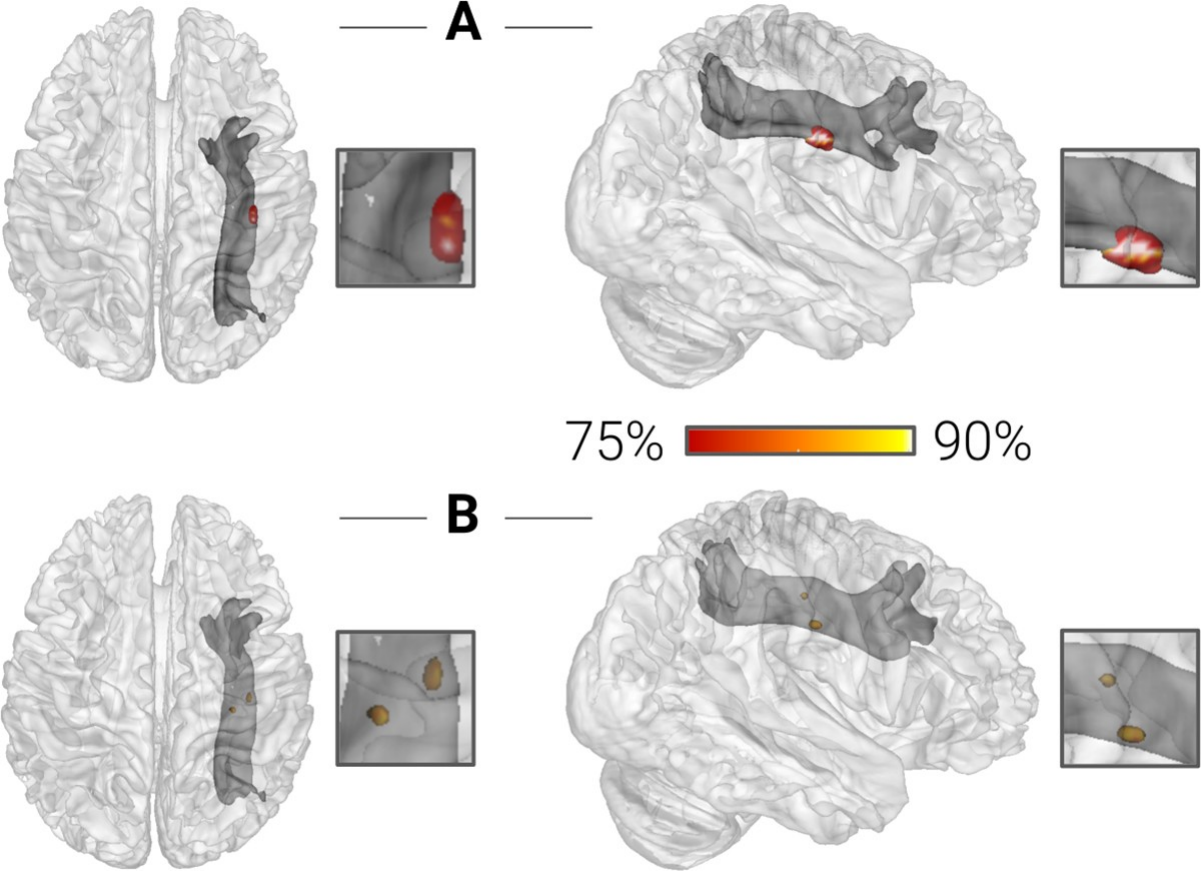
Structural connectivity classification results. The color bar indicates the peak accuracy for the classification of children with dyscalculia versus typical controls (*P* < 0.001, corrected by permutation testing) based on the structural connectivity (streamline density) of the right SLF connecting the right PPC and the right DLPFC. The numerical data used in this figure are included in S4 Data. DLPFC, dorsolateral prefrontal cortex; PPC, posterior parietal cortex; SLF, superior longitudinal fasciculus.

Finally, we examined differences in structural connectivity within the right SLF, the WM fiber tract connecting the PPC and the DLPFC [6]. In this analysis, we focused on a streamline density index since our searchlight-based multivariate pattern analysis approach required a three-dimensional tract reconstruction as a search space. The classification of children that developed dyscalculia versus typically developing children yielded a peak accuracy of 77.78% when seeding from the PPC cluster derived from the amplitude of low-frequency fluctuations and a peak accuracy of 81.48% when seeding from the PPC cluster derived from regional homogeneity (all *P* < 0.001, corrected by permutation testing) (Fig 4, Table 4).

**Table 4:** Structural connectivity classification results.

**Streamline density of the SLF** ^**1**^								
**Size (voxels)**	**Size (mm** ^**3**^ **)**	**Peak accuracy (%)**	**Sensitivity at peak**	**Specificity at peak**	**MNI coordinates xyz**			**Anatomical location**
46	46	77.78	86.67	66.67	+25	−15	+37	SLF
**Streamline density of the SLF** ^**2**^								
**Size (voxels)**	**Size (mm** ^**3**^ **)**	**Peak accuracy (%)**	**Sensitivity at peak**	**Specificity at peak**	**MNI coordinates xyz**			**Anatomical location**
10	10	81.48	86.67	75.00	+18	−17	+34	SLF
17	17	77.67	93.34	58.34	+35	−14	+26	SLF

^1^ Cluster derived from the amplitude of low-frequency fluctuations.

^2^ Cluster derived from regional homogeneity.

MNI, Montreal Neurological Institute; SLF, superior longitudinal fasciculus.

## Discussion

In this study, we investigated the neurodevelopmental predisposition for dyscalculia using functional and structural MRI data of 3- to 6-year-old children without formal mathematical learning experience who either went on to develop the disorder or not. These 2 groups were significantly differentiated based on the regional functional activity of the right PPC, the network-level functional activity of the right DLPFC, and the effective functional and structural WM connectivity of both regions.

### Role of the frontoparietal system for typical and atypical mathematical learning

Right PPC dysfunction is a prime candidate for dyscalculia since this region is known to underlie visuospatial numerosity detection in early childhood and possibly already in infancy [13–15]. Our data confirm this notion and provide evidence for a dissociation between the prediction of numerosity detection skills from parietal cortex activity and the prediction of calculation problem solving skills from prefrontal cortex activity. This dissociation is supported by single-cell recordings in nonhuman primates showing that parietal neurons respond significantly earlier to numerosity than prefrontal neurons [16,17]. Accordingly, the prefrontal cortex is thought to receive numerosity information from the parietal cortex for further goal-directed processing [16,17]. Our effective connectivity results suggest that a reduction of this parietal-to-frontal numerosity information transfer is a central neurobiological predisposition for developing dyscalculia. At the same time, our data indicate that, to a lesser degree, the frontal-to-parietal retrieval of this information also seems to be hampered in children that are predisposed for dyscalculia. While these interpretations remain to be verified in a task-based fMRI experiment, they provide a plausible possible explanation for the hallmark behavioral feature that individuals with dyscalculia need much more time than unaffected individuals to solve mathematical problems. Alternatively, it is also possible that the observed effects could be due to group differences in attention or working memory, cognitive systems that are also implemented in parietal and prefrontal cortices [18,19]. In this case, follow-up task-based fMRI studies are necessary to determine whether number-specific or domain-general attention and working memory systems are affected in dyscalculia.

A central portion of the SLF, the structural backbone of frontotemporal signal transfer, revealed streamline density differences between future dyscalculics and controls. This observation is in line with a study that found an association between the fractional anisotropy of a central part of this fiber tract and calculation problem solving in 10- to 15-year-old children [6]. Future longitudinal work in even younger children will have to clarify whether these structural connectivity differences precede the reported functional connectivity differences or vice versa.

### Right hemisphere lateralization of neurodevelopmental differences in dyscalculia

A recent meta-analysis revealed that children with a mean age of 9 years show a right-lateralized parietal cortex response when solving numerosity tasks and a left-lateralized parietal cortex response when solving calculation tasks [5]. The right parietal cortex differences we found fit into this picture given that the 3- to 6-year-old children studied here have at best vestigial calculation experience. A meta-analysis in adults suggests that brain responses during numerosity and calculation tasks become bilaterally distributed over parietal and prefrontal cortices toward adulthood [20]. A possible explanation for this developmental expansion to the left parietal cortex could be that adults rely on verbalized numerosity fact retrieval, a strategy that may still be developing in children. Verbal numerosity processing in the left parietal cortex thus may not be a predisposition for dyscalculia, but a consequence of deficient nonverbal numerosity processing. A long-term longitudinal study would be necessary to corroborate this hypothesis.

### Predictive performance in comparison to behavioral screening benchmarks

With a peak classification accuracy of 87%, the best MRI-based early predictors of dyscalculia reported in this study outperformed the best behavioral early screening tools currently available for which accuracy rates of 76% to 79% are documented [21]. This result is remarkable when contrasting the short acquisition time of the resting-state fMRI scan used in our study (3 minutes and 24 seconds) with typical behavioral test durations of 30 to 40 minutes. Furthermore, our MRI-based approach in principle works for children as young as 3 years, whereas current behavioral test instruments can only be used a few months before school at 5 to 6 years of age.

### Operational definition of dyscalculia and group matching for cognitive skills

It must be noted that the operational definition of dyscalculia used here (1 SD below the math performance of a reference population) is more lenient than the definitions used in the DSM-5 (1.5 SDs) and the ICD-11 (2 SDs). Nevertheless, we assume that this diagnostic discrepancy does not compromise the generalizability of the present findings to more severely affected individuals. The rationale for this assumption is that there is a large body of genetic, neural, and behavioral evidence suggesting that dyscalculia forms the lower end of a continuum of math ability rather than a qualitatively distinct ability profile [22,23]. That being said, since math ability was assessed only once at the end of second grade in school, we could not ensure the persistence of math difficulties and thus not exclude an inflated false-positive rate.

While the 2 groups were matched for cognitive skills based on behavioral assessment data collected at a preschool age, we acknowledge that the 2 groups might not be balanced anymore in second grade with respect to language ability, nonverbal IQ, and verbal short-term memory. Nevertheless, the currently available literature suggests that possible differences between affected and typically developing children can be expected to reach small or at best moderate effect sizes [23].

### Statistical power

It is important to point out that published effect sizes of neuroimaging data tend to be overestimated. This limitation also applies to the 2 studies included in the power analysis conducted for the present study. Therefore, we have to acknowledge that the large power levels reported here might have been overestimated. In any case, we cannot assume that the present study is sufficiently powered to also detect moderate or small effects. Additionally, our power analysis is based on a univariate statistical framework that can only indirectly inform but not fully capture the complex multivariate pattern analyses conducted here.

## Conclusions

The present study identified a neurobiological early childhood predisposition for dyscalculia characterized by altered spontaneous activity, functional interaction, and structural connectivity of a frontoparietal network in the developing brain.

## Methods

### Participants

Participants were recruited between 2012 and 2013 from the Leipzig metropolitan area as part of a larger study (82 children) focusing on developmental learning disorders. Participants were partially preselected by specifying children with a familial risk for learning difficulties as the main target group during recruitment. An initial screening revealed 54 children who did not have a history of neurological, psychiatric, hearing or vision disorders, that they were native German monolingual speakers, and that they did not yet receive math instruction. In this context, it has to be noted that in many European countries, including Germany, typical state kindergartens are not part of the school system and do not provide formal math education. MRI data recording and behavioral testing took place between 2013 and 2014 at a kindergarten age of 3 to 6 years. Follow-up math assessment was conducted between 2017 and 2018 at the end of second grade in school when the children were 7 to 9 years old. All parents gave written informed consent, and all children gave verbal informed assent to participate. The study was approved by the Ethics Committee of the University of Leipzig, Germany (approval number 320-11-26092011).

### Operational definition of developmental dyscalculia

Dyscalculia was operationally defined at the end of second grade based on a standardized and age-normed mathematical ability test. Children were assigned to the dyscalculia group (*n* = 15) if they performed equal to or below the 16th percentile rank of the reference population performance (equivalent to 1 SD below the mean of the normal distribution or a T-score of 40). These 15 children were matched to a control group of 15 children who performed equal to or above the 30th percentile rank in the math test, but did not differ in terms of age, sex, handedness, maternal education, nonverbal IQ, and verbal short-term memory. The matching was done with R–3.6.3 (https://www.r-project.org) by randomly sampling groups of 15 control children from a pool of 39 possible candidates 500 times, generating 500 unique matchings. For each of these random matchings, statistical differences between the dyscalculia group and the prospective control group with respect to the abovementioned covariates were assessed. The matching that most effectively minimized differences between groups (i.e., maximized the *P* values of the statistical comparisons) was selected to derive the control group. None of the participants in the final sample scored below 70 in a nonverbal IQ test (as required by the ICD-11) or received a diagnosis of developmental dyslexia or attention deficit hyperactivity disorder (according to parental questionnaires).

A priori power analyses were based on the framework of the difference between 2 independent means implemented in the software package G*Power (http://www.gpower.hhu.de). The effect sizes of the previous studies used for these power analyses were derived by *z*-transforming the reported peak *t*-statistics for particular regions of interest and use the resulting *z*-scores to compute Cohen’s *d* based on the formula described in [24].

### Standardized behavioral testing

Handedness was assessed with a customized version of the Edinburgh Handedness Inventory that was adapted for children. The participants were asked to perform or simulate everyday activities with their hands so that we were able to calculate a laterality quotient (LQ). Right-handedness was defined as LQ > +48, left-handedness as LQ ≤ –28, and ambidexterity as –28 < LQ ≤ +48.

Maternal education was assessed with an in-house questionnaire and defined as the sum of school education and professional education. School education was quantified on a scale from 0 to 3 (0 = no school graduation, 1 = graduation after 9 years (German “Hauptschulabschluss”), 2 = graduation after 10 years (German “Mittlere Reife”), and 3 = high school graduation). Higher education was quantified on a scale from 0 to 4 (0 = no professional degree, 1 = vocational degree, 2 = university of applied sciences degree, 3 = college graduate, and 4 = graduate degree).

To derive a nonverbal IQ score, we used the perceptual reasoning subscale of the Wechsler Intelligence Scale for Children (WISC-IV) (https://www.testzentrale.de/shop/wechsler-intelligence-scale-for-children-fourth-edition.html).

Mathematical ability was assessed using the Heidelberg calculation test (https://www.testzentrale.de/shop/heidelberger-rechentest.html). This comprehensive tool consists of 11 subtests covering addition, subtraction, multiplication, division, symbolic and nonsymbolic numerosity comparison, numerosity estimation, numerical sequencing, and counting. Correct answers were added together and transformed into a percentile rank based on age norms for a full scale of total mathematical ability comprising 2 subscales: visuospatial numerosity detection (including symbolic and nonsymbolic numerosity comparison, numerosity estimation, numerical sequencing, and counting) and calculation problem solving (including addition, subtraction, multiplication, and division).

Handedness and intelligence were assessed individually in a single session in a small child laboratory room. Mathematical ability was assessed as a group test (max. 15 children) in a separate session in a larger seminar room. In each sample, these data were acquired by max. 3 different research assistants that were thoroughly familiarized with the testing procedure beforehand. Before collecting the data, each assistant passed 3 supervised practice sessions with children that were not enrolled in the current study.

### Magnetic resonance imaging

MRI data were acquired on a 3T Trio scanner (Siemens, Erlangen, Germany) with a 12-channel head coil. T1-weighted MP2RAGE images were recorded with the parameters TR = 5,000 ms, TE = 2.82 ms, TI = 700 ms, matrix size: 250 × 219 × 188 and 2 different voxel sizes (10 cases / 10 controls: 1.3 × 1.3 × 1.3 mm; 5 cases / 5 controls: 1.0 × 1.0 × 1.0 mm). T2*-weighted EPI images (100 resting-state fMRI volumes) were recorded with the parameters TR = 2,000 ms, TE = 30 ms, voxel size 3 × 3 × 3.9 mm, matrix size: 192 × 192 × 111. Diffusion-weighted EPI images (60 diffusion-encoding directions) were acquired with the parameters TR = 8,000 ms, TE = 83 ms, voxel size 1.9 × 1.9 × 1.9 mm, matrix size: 192 × 192 × 111. Participants underwent an extensive training session in a mock scanner prior to the actual scanning session. During the 30-minute training, children received continuous real-time feedback regarding their head motion via a motion sensor system.

### T1-weighted data processing

T1-weighted MP2RAGE images were first visually inspected to ensure that the data were not corrupted by imaging artifacts including diffuse image noise along the phase encoding direction, ghosting, or Gibbs artifacts. Subsequently, images were skull-stripped and aligned with a template derived from a reference sample of 4.5- to 8.5-year-old children (http://www.bic.mni.mcgill.ca/ServicesAtlases/NIHPD-obj1) with an isotropic resolution of 1.0 mm in Montreal Neurological Institute (MNI) space using Freesurfer Version 5.3.0 (http://surfer.nmr.mgh.harvard.edu). We then normalized the images to an age-specific template in MNI space that we directly derived from the present sample using Advanced Normalization Tools Version 2.2.0 (http://picsl.upenn.edu/software/ants). Due to the differences with respect to the original voxel sizes, the T1 data were only used for normalizing the fMRI data and not for morphometric analyses. All anatomical locations were identified with the Harvard–Oxford Cortical Structural Atlas implemented in FSL 5.0.9 (https://fsl.fmrib.ox.ac.uk).

### T2*-weighted resting-state fMRI data processing

T2*-weighted resting-state fMRI data were preprocessed using FSL 5.0.9 and MATLAB R2017b (https://www.mathworks.com). After removing the first 4 volumes of each scan, data were slice-time corrected. Head motion was quantified by framewise displacement (the sum of rotational and translational rigid body realignment parameters from one volume to the next) [25]. To account for head motion, volumes with a framewise displacement >0.5 mm were removed from further analysis. The number of volumes remaining did not differ significantly between the dyscalculia group (mean: 83, SD: 17) and the control group (mean: 88, SD: 10) (Cohen’s *d* = 0.10, *P* = 0.574).

Partial volume maps for gray matter (GM), white matter (WM), and cerebrospinal fluid (CSF) were generated from the segmented MNI T1 data (FSL FAST). WM and CSF masks were thresholded at 80% tissue probability, before rigid alignment to individual resting-state fMRI space. To further control for head motion as well as scanner-related and physiological noise, 5 principal components from WM and CSF were regressed out together with the 6 linearly detrended motion parameters [26]. Residual data were band-pass filtered at 0.01 to 0.1 Hz and spatially smoothed with a 6-mm full width at half maximum (FWHM) kernel, leading to an effective smoothness of around 8 to 9 mm.

In the final step, we computed regional homogeneity values, the fractional amplitude of low-frequency fluctuations and degree centrality at the whole-brain level [27–29]. Regional homogeneity quantifies the coherence of hemodynamic time series within a confined neighborhood of voxels. Specifically, it is defined as Kendall’s coefficient of concordance [30] of a given voxel with the voxels within its immediate vicinity. The index may range from 0 to 1, with higher values indicating that the time series of the examined neighborhood of voxels is temporally more homogeneous. The fractional amplitude of low-frequency fluctuations corresponds to the ratio (i.e., the contribution) of the low-frequency amplitude (i.e., 0.01 to 0.08 Hz) to the amplitude to the entire frequency range (i.e., 0 to 0.25 Hz) [30]. To obtain the fractional amplitude of low-frequency fluctuations for a given voxel, the frequency spectrum of the preprocessed data is determined first and then the sum of the amplitude across the whole frequency spectrum along with the amplitude over the low-frequency range are computed before the ratio is taken in the final step. Regional homogeneity and the fractional amplitude of low-frequency fluctuations were computed with the DPARSF toolbox (http://rfmri.org/DPARSF) in SPM version 12.7219 (https://www.fil.ion.ucl.ac.uk/spm/ software/spm12/). Degree centrality quantifies the similarity of the hemodynamic time series within a voxel to the time series of all other voxels across the whole brain. Degree centrality was computed with in-house code based on the *3dTcorrMap* utility implemented in AFNI (https://afni.nimh.nih.gov/).

### Diffusion-weighted data processing

Diffusion-weighted imaging data were available for 29 out of 30 children. Prior to preprocessing, these data were semiautomatically and visually inspected for motion artifacts by identifying signal dropouts [31]. This inspection revealed that 2 children had to be excluded due to insufficient data quality (i.e., signal dropout in superior and temporal regions). Accordingly, the final sample for this analysis comprised 13 children with dyscalculia and 14 controls. Further preprocessing was performed using FSL. Data were corrected for motion by affinely aligning volumes with different b-values to respective averages previously rigidly aligned with the individual participant’s MNI T1 image. Subsequently, the diffusion tensor (FSL DTIFIT) and the fiber orientation distribution for each voxel were determined (FSL BEDPOSTX).

Tractograms were computed by applying probabilistic tractography (FSL PROBTRACKX2). Region of interest (ROI) masks of target tracts were generated by using each cortical ROI involved, once as seed region and once as target region. We seeded 5,000 streamlines (curvature threshold = 0.2, step length = 0.5 mm) in each voxel within the GM–WM interface of the seed region at hand. Tracking was restricted using a rectangular ventral exclusion mask to quantify structural connectivity of the right PPC and the right DLPFC via the SLF. The corresponding mask was defined within the common MNI group template covering the entire x-y-plane at z = 10 and then aligned with the individual T1 map in MNI space.

Resulting streamline density maps were first log-transformed and then voxel-wise divided by the log-transformed maximal number of possible streamlines. Summed, log-transformed and normalized maps were averaged and thresholded at the 80th percentile to extract only the core part of the respective tract.

### Classification analysis

A searchlight-based multivariate pattern analysis approach was used to identify voxels that separated individuals with dyscalculia from controls significantly above chance. In contrast to conventional univariate analyses, multivariate pattern analysis is known to yield a more sensitive and specific classification since signals from individual voxels are jointly analyzed so that a richer information structure can be decoded. If, for example, differences between groups are encoded in distributed patterns of activity rather than isolated voxel-level activity, multivariate pattern analysis is able to distinguish between these groups, while univariate approaches will not be able to uncover this spatially more complex information. This analysis was implemented such that for each voxel within the entire GM or a certain WM fiber tract we defined a spherical surrounding region (the searchlight) with a radius of 4 mm (including 7 voxels, 189 mm^3^) and performed support vector classification analyses for each possible searchlight position within a 10-fold cross validation design. The cross-validation was stratified to avoid an imbalance between the numbers of subjects of each group across folds. Per fold, 3 participants were selected to be used for testing, while the remaining 27 data sets served for training. Sensitivity, specificity and accuracy were assigned to each voxel at its center and nonparametrically assessed for significance by running 10,000 permutations of the training and test data (group labels) to yield a voxel-wise null distribution. During the permutation test correction for false positives, the observed results were randomly resampled 10,000 times to build an empirical estimate of the null distribution to draw the classification accuracy from. Voxels were identified as significant by counting the number of times the accuracy was smaller or greater than the accuracy value obtained from the permuted data sets, and multiplying this value by the minimal *P* value of the permutation test (1/(n+1), *n* = 10,000). These analyses were carried out using The Decoding Toolbox Version 3.999 (https://sites.google.com/site/tdtdecodingtoolbox) and MATLAB 2017b.

### Regression analysis

Using the same software tools and parameters for the searchlight, the cross-validation, and the permutation as in the classification analyses described above, we also conducted searchlight-based support vector regression analyses. The goal of these analyses was to compute voxel-wise (not subject-wise) coefficients of determination (*R*^*2*^) to identify significant associations between the fMRI data and subscale test scores driving the classification results in a certain cluster. Regression models were run individually for each voxel within the region of interest before comparing the distributions. Distributions of the coefficients of determination were compared between groups by running either Student *t* tests (normally distributed data) or Wilcoxon signed-rank tests (not normally distributed data) using R–3.6.3.

### Effective connectivity analysis

To examine the effective connectivity between pairs of regions of interest, we determined the linear directional influence of one hemodynamic time series onto the other (Granger causality). Specifically, we used a signed path coefficient of linear regression framework implemented in the REST-GCA toolbox (http://www.restfmri.net). In this framework, positive and negative path coefficients reveal whether the preceding activity of a region predicts the increased or decreased present activity of another region. Granger causality indices were compared between groups by running either Student *t* tests (normally distributed data) or Wilcoxon signed-rank tests (not normally distributed data) using R–3.6.3.

## Supporting information

S1 Data Numerical values (peak classification accuracy) related to the brain image in Fig 1. (XLSX)

S2 Data Numerical values (peak classification accuracy) related to the brain image in Fig 2. (XLSX)

S3 Data Numerical values (Granger causality indices) related to the box plots in Fig 3. (XLSX)

S4 Data Numerical values (peak classification accuracy) related to the brain image in Fig 4. (XLSX)

## Abbreviations

CSF: cerebrospinal fluid
DLPFC: dorsolateral prefrontal cortex
FWHM: full width at half maximum
GM: gray matter
IQ: intelligence quotient
LQ: laterality quotient
MNI: Montreal Neurological Institute
MRI: magnetic resonance imaging
PC: precuneus
PPC: posterior parietal cortex
ROI: region of interest
SD: standard deviation
SLF: superior longitudinal fasciculus
WISC-IV: Wechsler Intelligence Scale for Children
WM: white matter
